# Dimethyl Fumarate Was Ineffective but Not Harmful for a Patient with Myelin Oligodendrocyte Glycoprotein Antibody Disease

**DOI:** 10.7759/cureus.6040

**Published:** 2019-10-30

**Authors:** Yoko Warabi, Toshiyuki Takahashi, Eiji Isozaki

**Affiliations:** 1 Department of Neurology, Tokyo Metropolitan Neurological Hospital, Tokyo, JPN; 2 Department of Neurology, Tohoku University Graduate School of Medicine and National Hospital Organization Yonezawa National Hospital, Sendai, JPN

**Keywords:** myelin oligodendrocyte glycoprotein, mog, myelin oligodendrocyte glycoprotein antibody disease, mog antibody disease, dimethyl fumarate, neuromyelitis optica spectrum disorders, multiple sclerosis, disease modifying drug

## Abstract

We treated a myelin oligodendrocyte glycoprotein (MOG) antibody disease patient who had been prescribed dimethyl fumarate because she was thought to have been suffering from multiple sclerosis (MS). Mild optic neuritis relapsed at one year and four months after the administration of dimethyl fumarate. Therefore, dimethyl fumarate was ineffective for preventing relapse of MOG antibody disease. However, dimethyl fumarate for MOG antibody disease was not harmful compared with when disease-modifying drugs (DMDs) of MS were used for anti-aquaporin-4 antibody-positive neuromyelitis optica. If MS patients repeat relapses even after the start of DMDs, a differential diagnosis including MOG antibody disease should be made.

## Introduction

The typical clinical picture of myelin oligodendrocyte glycoprotein (MOG) antibody disease is known as neuromyelitis optica spectrum disorders (NMOSD), acute disseminated encephalomyelitis in childhood or optic neuritis [1, 2]. Anti-aquaporin-4 (AQP4) antibody is not present in MOG antibody disease patients' serum, and relapses of MOG antibody disease are often milder than those of anti-AQP4 antibody-positive NMOSD. If acute myelitis with longitudinally extensive transverse myelitis was not experienced, MOG antibody disease patients cannot satisfy the diagnostic criteria for NMOSD with negative or unknown AQP4-IgG antibody status [3]. If multiple white matter lesions are relapsing in the cerebrum, MOG antibody disease cannot be distinguished from multiple sclerosis (MS) without anti-MOG antibody measurement [4]. MOG antibody disease patients may satisfy the diagnostic criteria of MS, such that disease-modifying drugs (DMDs) for MS are prescribed to MOG antibody disease patients. It has been reported that interferon-beta, fingolimod, natalizumab and dimethyl fumarate, which are DMDs for MS, can cause severe disease exacerbation when used for anti-AQP4 antibody-positive NMOSD [5-9]. However, there is little knowledge on the effect and safety of DMDs for MS on MOG antibody disease [10].

## Case presentation

**Figure 1:**
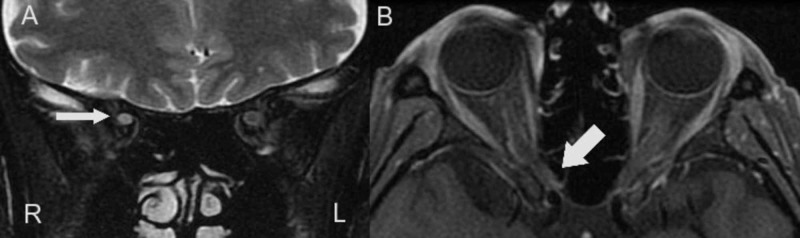
Optic nerve MRI findings. T2-weighted coronal imaging showed that the right optic nerve had atrophied (A). T1-weighted axial imaging showed that the right optic nerve was actively enhanced near the apex of the orbit region (B).

Our female patient initially developed right optic neuritis at the age of 44 years with right eye pain and blurred vision. Head MRI revealed the right optic neuritis and a contrast-enhanced white matter lesion around the left lateral ventricle. Anti-AQP4 antibody was absent in her serum. She was diagnosed with a clinically isolated syndrome of MS. Her vision recovered with intravenous methylprednisolone (IVMP) therapy. At the age of 45 years, the right optic neuritis relapsed. Her vision recovered again with IVMP therapy. At the age of 50 years, she felt weakness and tightness of the right lower limb. Contrast-enhanced lesions in the bilateral corona radiata were seen in MRI. Her illness was diagnosed as MS, and remission was achieved with IVMP therapy. At the age of 51 years, she underwent IVMP therapy because of dullness of the right lower limb, but the symptom mildly remained as Expanded Disability Status Scale of Kurtzke (EDSS) 1.5. At the age of 52 years, she had difficulty in climbing the stairs because of the weakness of her right lower limb. She had a relapse of MS derived from a short white matter lesion in the fourth cervical spinal cord. IVMP therapy was not effective, and symptoms slightly improved with immunoadsorption plasmapheresis, but sequelae remained as EDSS 3.0. She was prescribed dimethyl fumarate as a DMD for MS. The only adverse effect of dimethyl fumarate was occasional mild nausea. However, mild optic neuritis relapsed at one year and four months after the start of dimethyl fumarate, at the age of 53 years, and she was hospitalized in our hospital for the first time. Neurological examination revealed pain and central scotoma in the right eye, deep tendon hyperreflexia of the right upper and lower limbs, mild weakness of the right iliopsoas muscle, walking with dragging of the right foot, impossibility of tandem gait or one foot standing and a decreased vibratory sensation of the left lower limb. In head MRI, the right optic nerve had atrophied and was actively enhanced near the apex of the orbit region (Figure 1 A, 1B).

**Figure 2:**
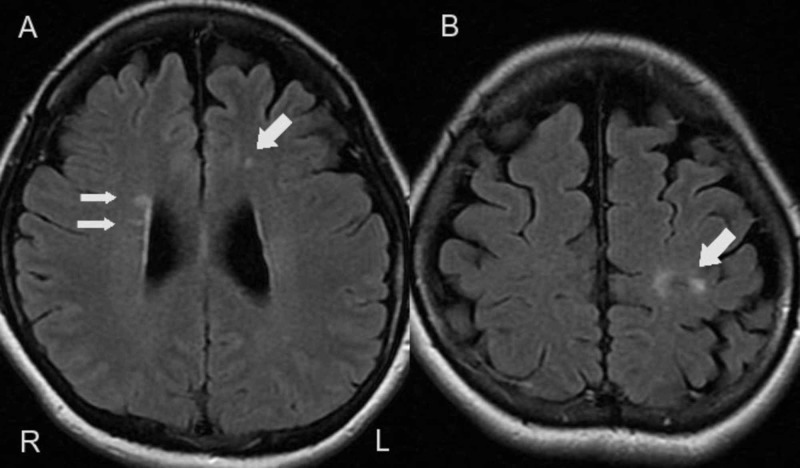
Brain MRI findings. In the cerebrum, fluid attenuation inversion recovery axial imaging showed that multiple white matter lesions were presented around the lateral ventricles (A) and juxtacortical region (B).

In the cerebrum, multiple white matter lesions were presented around the lateral ventricles and juxtacortical region, but at this time the contrast enhancement effect was negative (Figure 2A, 2B).

**Figure 3:**
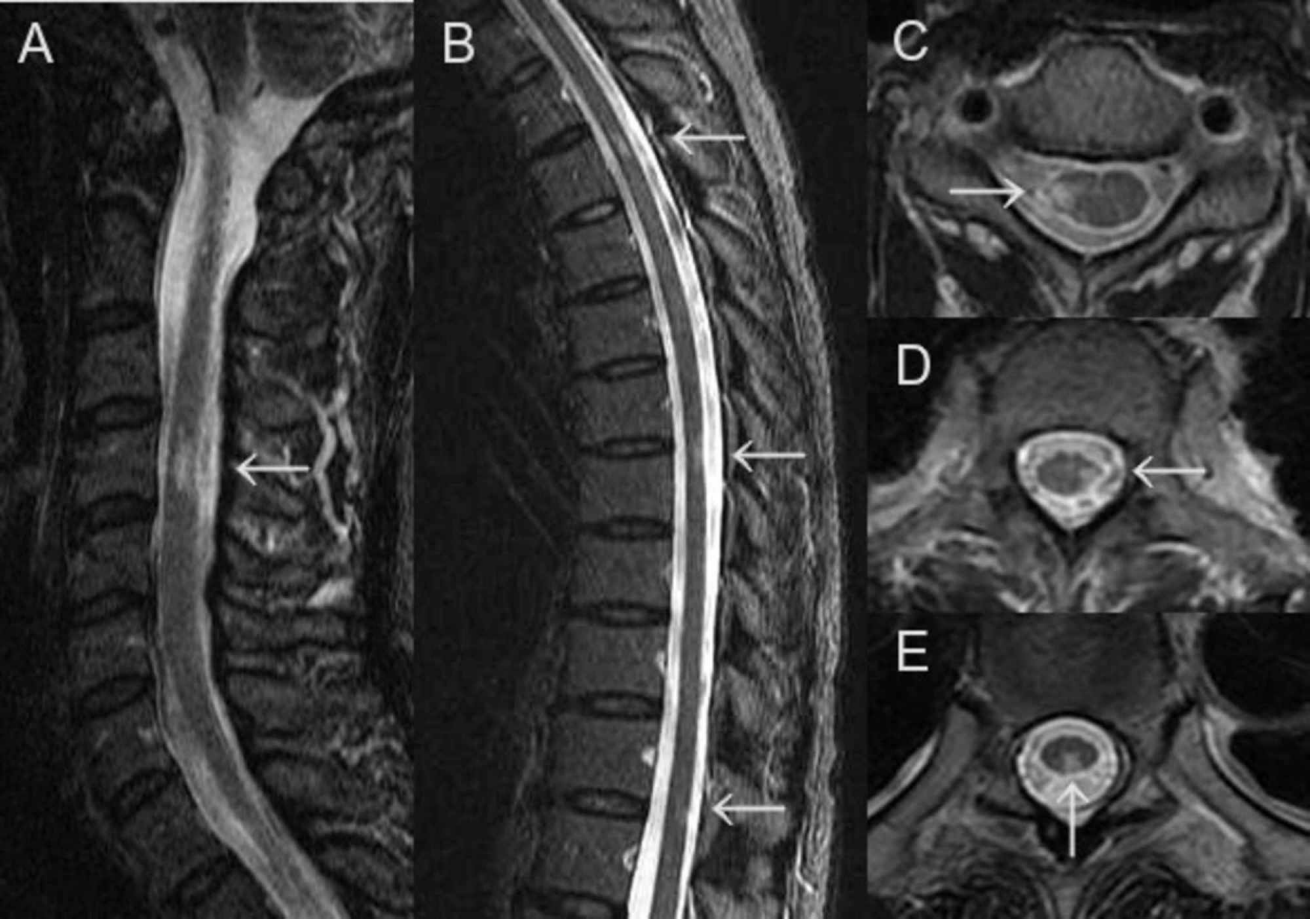
Spinal cord MRI findings. In spinal cord MRI, old short lesions of the cervical (A) and thoracic (B) spinal cord were observed at the central part in C3/4, the right side in C4 (C), the left lateral funiculus in T1/2 (D), the central part and posterior funiculus from T4 to T5, the posterior funiculus in T7/8 (E) and the posterior funiculus in T11/12.

In spinal cord MRI, old short lesions of the cervical (C) and thoracic (T) spinal cord were observed at the central part in C3/4, the right side in C4, the left lateral funiculus in T1/2, the central part and posterior funiculus from T4 to T5, the posterior funiculus in T7/8 and the posterior funiculus in T11/12 (Figure 3A-3E).

**Figure 4:**
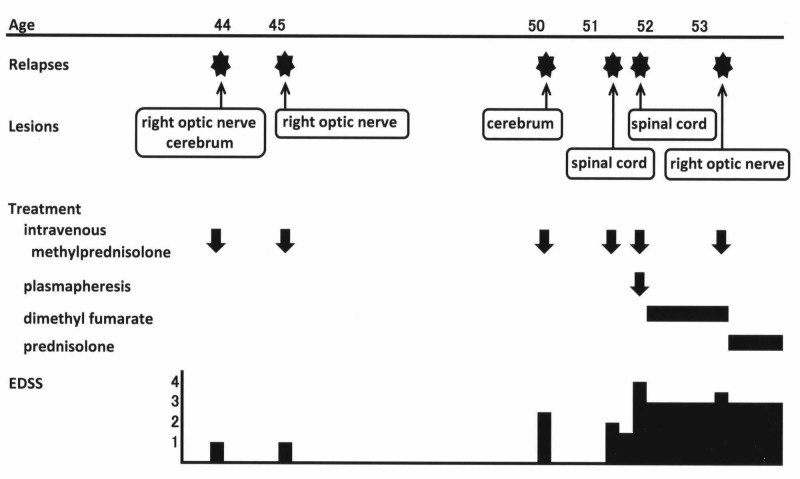
Summary of clinical course. Onset of MOG antibody disease was at 44 years old. Optic neuritis, myelitis and cerebral white matter lesions relapsed. Mild optic neuritis relapsed at one year and four months after the start of dimethyl fumarate. Therefore, dimethyl fumarate was ineffective for preventing relapses of MOG antibody disease. However, dimethyl fumarate was not harmful for our MOG antibody disease patient because it did not increase the frequency or severity of relapse. EDSS, Expanded Disability Status Scale; MOG, myelin oligodendrocyte glycoprotein.

A speckled pattern of positivity for antinuclear antibody and anti-MOG antibody was present in her serum. Anti-SS-A and anti-SS-B antibodies, HTLV-1 antibody and anti-thyroid peroxydase antibody were negative in her serum. Cerebrospinal fluid examination did not detect oligoclonal IgG bands. Active right optic neuritis was the diagnosis, and her right eye scotoma disappeared by IVMP therapy. Critical flicker-fusion frequency recovered as normal as 36 Hz. The diagnosis of her illness was changed from MS to MOG antibody disease. She stopped dimethyl fumarate therapy and switched to oral prednisolone (10 mg/day) (Figure 4).

## Discussion

Dimethyl fumarate therapy for MOG antibody disease was not harmful compared with when DMDs of MS were used, incidentally, for anti-AQP4 antibody-positive NMOSD. Dimethyl fumarate did not increase the frequency or severity of relapse of MOG antibody disease in our patient. It has been reported that interferon-beta, fingolimod, natalizumab and dimethyl fumarate, which are DMDs for MS, can cause severe disease exacerbation when used for anti-AQP4 antibody-positive NMOSD [5-9]. Anti-AQP4 antibody-positive NMOSD patients who developed catastrophic relapses following initiation of dimethyl fumarate were reported [9]. These patients developed severe myelitis extending from the cervical cord to the medulla with significant cord swelling, resulting in complete quadriplegia and respiratory difficulties, in addition to severe bilateral visual loss. Although there are few reports of using DMDs of MS for MOG antibody disease patients (Table 1), reported effects of interferon-beta for MOG antibody disease were various from successfully effective to marked disease exacerbation [10, 11]. Glatiramer acetate showed decrease or increase in relapse rate, and natalizumab showed remarkable increase in relapse rate for MOG antibody disease patients [10]. Fingolimod for MOG antibody disease has one report with remarkable deterioration [12].

**Table 1:** Reports of using DMDs of MS for MOG antibody disease patients DMDs: disease-modifying drugs MS: multiple sclerosis MOG: myelin oligodendrocyte glycoprotein

Author/publication year	DMD	Effect
Xu et al. [11] (2012)	interferon-beta	successfully effective
Jarius et al. [10] (2016)	interferon-beta	no decrease in relapse rate and marked disease exacerbation
Jarius et al. [10] (2016)	glatiramer acetate	decrease or increase in relapse rate
Jarius et al. [10] (2016)	natalizumab	remarkable increase in relapse rate
Miyazaki et al. [12] (2016)	fingolimod	remarkable deterioration
This case	dimethyl fumarate	did not increase the frequency or severity of relapse

For our MOG antibody disease patient, dimethyl fumarate seemed to be unharmful. Because this patient had relapses more than once a year for the past two years, even though one relapse occurred at one year and four months after the start of dimethyl fumarate, we suspect that her relapse frequency after the start of dimethyl fumarate did not increase significantly (Figure 4). Asymptomatic cerebral white matter lesions also did not increase. Dimethyl fumarate did not increase the severity of relapse of MOG antibody disease because her optic neuritis was mild, and critical flicker-fusion frequency recovered as normal. There was no severe adverse effect of dimethyl fumarate. Thus, although dimethyl fumarate was ineffective for relapse prevention, it seemed to be unharmful and did not increase the frequency or severity of relapse of MOG antibody disease. However, even though we report that dimethyl fumarate is not harmful, there is a possibility that other patients will show other effects, as in past reports on interferon-beta and glatiramer acetate therapy for MOG antibody disease. Therefore, dimethyl fumarate cannot be recommended for MOG antibody disease.

If multiple white matter lesions are relapsing in the cerebrum, MOG antibody disease cannot be distinguished from MS without anti-MOG antibody measurement [4]. Our patient showed periventricular and juxtacortical lesions, which are typical for MS, starting from when she experienced the first right optic neuritis. Her spinal cord lesions were all located in the white matter and were longitudinally short. Her optic neuritis was mild, and anti-AQP4 antibody was negative. Her diagnosis of MS had no contradiction. However, because MS and MOG antibody disease treatments are different, it is necessary to separate properly the diagnosis of MS from MOG antibody disease. In order to prescribe correct treatments to all patients, diagnostic criteria of MOG antibody disease and a measurement method for anti-MOG antibody should be established.

Although treatment for MOG antibody disease has not been established yet, about half of the cases of MOG antibody disease have been reported as having a monophasic course and no relapse [2]. As a therapy for acute relapse of MOG antibody disease, IVMP is often effective [13]. Prednisolone and immunosuppressants are often used for patients with repeated relapses [10]. Dimethyl fumarate therapy could be continued for MOG antibody disease because it appears to be unharmful. However, the patient’s health would be at risk because the wrong treatment is being prescribed. DMDs of MS have become increasingly effective, and no evidence of disease activity has become a goal for the treatment of MS [14]. However, if the diagnosis is not MS, DMDs will not work. Therefore, when MS patients have repeated relapses even after the start of DMDs, we believe that a differential diagnosis including MOG antibody disease should be made.

Dimethyl fumarate may exert immunomodulatory as well as cytoprotective effects via activation of nuclear factor (erythroid derived 2)-like 2 transcription factor-mediated or hydroxycarboxylic acid receptor 2-mediated signalling pathways [15]. Dimethyl fumarate treatment of patients with MS affects predominantly memory T cells accompanied by a shift in helper T cell populations, resulting in a shift toward anti-inflammatory responses [16]. Frequencies of Th1 cells were decreased, whereas those of Th2 cells were increased. Because of this mechanism, dimethyl fumarate may adversely affects anti-AQP4 antibody-positive NMOSD. However, the cerebrospinal fluid cytokine profile in the acute phase of MOG antibody disease was reported to be similar to anti-AQP4 antibody-positive NMOSD but clearly different from MS [17]. The reason why dimethyl fumarate did not worsen MOG antibody disease is unknown.

## Conclusions

Dimethyl fumarate was not effective in preventing relapses in MOG antibody disease. However, dimethyl fumarate therapy for MOG antibody disease was not harmful compared to when DMDs of MS were used for anti-AQP4 antibody-positive NMOSD. Furthermore, dimethyl fumarate did not increase the frequency or severity of relapse of MOG antibody disease. If MS patients have repeated relapses even after the start of DMDs, a differential diagnosis including MOG antibody disease should be made.
